# Immunotherapy in the perioperative management of esophageal squamous cell carcinoma: recent developments

**DOI:** 10.3389/fonc.2025.1611284

**Published:** 2025-09-25

**Authors:** Mengfei Sun, Pengjie Yang, Ting Yang, Jingjing Zhang, Hui Li, Yong Li, Benben Zhu

**Affiliations:** ^1^ College of Pharmacy, Inner Mongolia Medical University, Hohhot, China; ^2^ Department of Thoracic Surgery, Peking University Cancer Hospital (Inner Mongolia Campus)/Affiliated Cancer Hospital of Inner Mongolia Medical University, Hohhot, Inner Mongolia, China; ^3^ Department of Digestive Medicine, Zhungeerqi Central Hospital, Erdos, Inner Mongolia, China; ^4^ Department of Thoracic Surgery, National Cancer Center/National Clinical Research Center for Cancer/Cancer Hospital, Peking Union Medical College, Chinese Academy of Medical Sciences, Beijing, China; ^5^ Department of Pharmacy, Peking University Cancer Hospital (Inner Mongolia Campus)/Affiliated Cancer Hospital of Inner Mongolia Medical University, Hohhot, Inner Mongolia, China

**Keywords:** esophageal squamous cell carcinoma, perioperative immunotherapy, immune checkpoint inhibitors, clinical studies, mechanism

## Abstract

Esophageal cancer ranks among the most prevalent malignancies of the gastrointestinal tract. Esophageal squamous cell carcinoma (ESCC), accounting for approximately 90% of all esophageal cancer (EC) cases, represents the dominant pathological subtype. For locally advanced ESCC at clinical stages II-IVA, surgery-based multidisciplinary treatment remains the primary management strategy. Despite concerted efforts, long-term outcomes for ESCC patients remain suboptimal. Recent years have witnessed significant advancements in immunotherapy, with PD-1/PD-L1 inhibitors demonstrating promising efficacy across various malignancies, particularly in ESCC. This review synthesizes the current landscape of perioperative immunotherapy for resectable ESCC, emphasizing the role of immune checkpoint inhibitors in the perioperative setting. Additionally, it highlights unresolved challenges in ongoing clinical research and provides insights into future directions for ESCC immunotherapy.

## 1 Introduction

Esophageal cancer is one of the most common malignant tumors of the digestive tract, originating from the esophageal epithelium (1, 2). Esophageal cancer ranks as one of the most common malignancies globally, causing over 500,000 cancer-related deaths annually and the seventh leading cause of cancer deaths in the world (3). Incidence rates vary significantly across regions, with East Asia reporting the highest burden—twice the global average. Histologically, esophageal cancer is classified into two major subtypes: esophageal squamous cell carcinoma (ESCC) and esophageal adenocarcinoma (EAC) (4–6). Although EAC predominates in low-incidence areas like Europe and North America, ESCC constitutes 90% of esophageal cancer worldwide, with China harboring over half of these cases (7–9).

While surgery remains the cornerstone for resectable esophageal cancer, adjuvant strategies are essential given the dismal 5-year survival (<25%) in patients treated with surgery alone (10–12). Neoadjuvant therapy has emerged as a critical preoperative strategy for esophageal cancer, not only enhancing local control rates and resectability but also improving R0 resection rates and overall survival. The CROSS and NEOCRTEC 5010 studies suggest that compared with surgery alone, neoadjuvant chemoradiotherapy followed by surgery can significantly improve the R0 resection rate, pathological complete response (pCR) rate, and overall survival (OS) of patients. This has established the role of neoadjuvant chemoradiotherapy in the preoperative treatment of locally advanced resectable ESCC in both Western and Eastern countries (11, 12). Two recent studies have concurrently published long-term follow-up data: the CROSS trial’s 10-year analysis revealed a 13% survival benefit for patients receiving neoadjuvant chemoradiotherapy, while the NEOCRTEC 5010 study demonstrated a significant improvement in 5-year survival rates from 49.1% (surgery alone) to 59.9% (chemoradiotherapy + surgery) (13, 14). Despite these advances, significant room for improvement remains. In the CROSS trial, mortality from esophageal cancer in the chemoradiotherapy arm reached 47%, with recurrence rates of 28.6% versus 35.4% in the surgery-alone group. The dominant recurrence pattern was distant metastases, highlighting the need for intensified systemic therapy.

With the rapid advancements in immunotherapy, an increasing number of esophageal cancer patients are deriving clinical benefit from this treatment modality (15–17). Landmark trials like KEYNOTE-181 have propelled esophageal cancer into the immunotherapy era, with robust efficacy data emerging in both second-line and first-line settings (18, 19). Recent years have witnessed intensive exploration of neoadjuvant and adjuvant immunotherapy strategies, with studies such as PALACE-1 and SCALE-1 demonstrating promising outcomes (20, 21). These approaches have improved local tumor downstaging and R0 resection rates, reducing surgical complexity. However, radiotherapy-associated treatment-related non-cancer mortality remains a critical concern (22). Consequently, investigators are now exploring neoadjuvant immunotherapy combined with chemotherapy to evaluate efficacy without radiotherapy. This de-escalated approach may enhance safety by eliminating one treatment modality. The Chinese REVO trial compared carboplatin-paclitaxel plus camrelizumab (nICT) versus concurrent chemoradiotherapy (nCRT) in resectable locally advanced ESCC. Results showed comparable pathological complete response (pCR) rates between nICT and nCRT (40.6% vs. 35.7%) with superior safety profiles (≥Grade 3 adverse events: 22.0% vs. 31.8%) (23). Meanwhile, the complexity of multimodal therapy imposes psychological and economic burdens on patients, underscoring the importance of simplified, clinically feasible neoadjuvant protocols in real-world practice.

Against this backdrop, this review synthesizes the current landscape of perioperative immunotherapy for resectable esophageal squamous cell carcinoma (ESCC), highlights the role of immune checkpoint inhibitors in this setting, and discusses promising directions for future research.

## 2 Classification and synergistic mechanisms of combination treatment strategies

### 2.1 Immunotherapy

**Figure 1:**
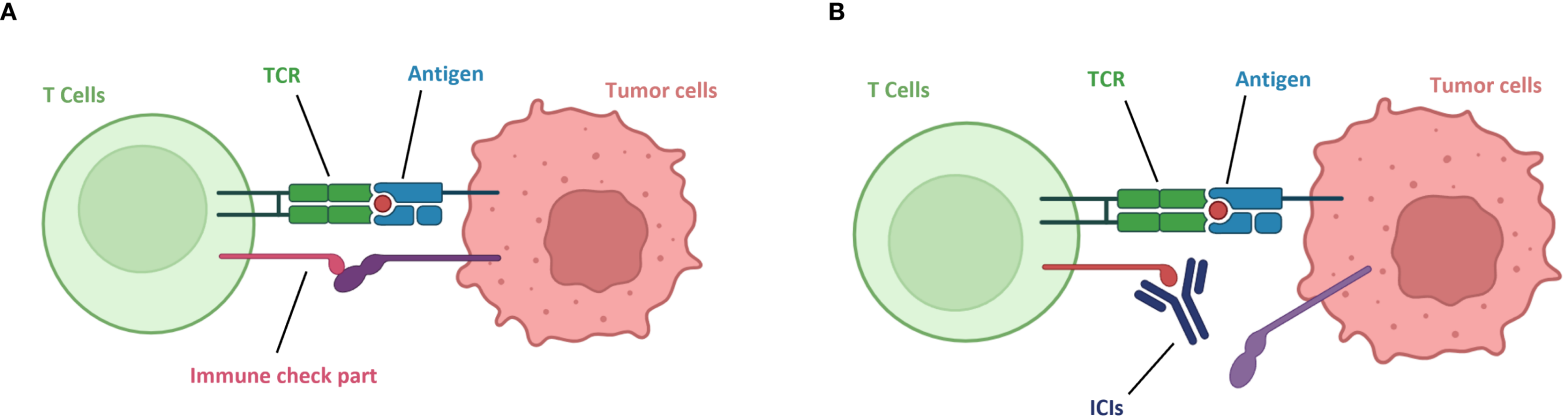
Mechanism of action of immune checkpoint inhibitors. **(A)** Tumor immune evasion. **(B)** Immune checkpoint inhibitors block tumor immune evasion, T cells re-recognize tumor cells.

Tumor cells primarily achieve immune evasion through low immunogenicity and induction of immune suppression. Some tumors lack protein peptides that can be presented by MHC or lose MHC molecules and fail to express co-stimulatory proteins, making them difficult to be recognized by the immune system. Meanwhile, tumor cells secrete immunosuppressive molecules such as TGF-β and IL-10, recruit regulatory T cells, and highly express immune checkpoint ligands, such as PD-L1, which binds to PD-1 on the surface of T cells to inhibit T cell activity. Immune checkpoint inhibitors specifically disrupt immune evasion. CTLA-4 inhibitors can block the inhibitory effect of the CTLA-4 protein, promote the differentiation of naïve T lymphocytes into mature T lymphocytes, and enhance the tumor-killing ability. PD-1/PD-L1 inhibitors, by blocking the binding of PD-1 to PD-L1, enable T lymphocytes to re-recognize tumor cells and restore the immune system’s attack on tumor cells, thereby exerting an anti-tumor effect (24, 25) (**Figure 1**).

### 2.2 Immunotherapy combined with chemotherapy

In recent years, clinical studies comparing neoadjuvant immunotherapy combined with chemotherapy to chemotherapy alone have been continuously carried out in esophageal squamous cell carcinoma (ESCC) and other cancers, and have demonstrated positive short-term therapeutic effects and long-term survival benefits. Researchers have explored the mechanism of action of the combination treatment of the two. Previous studies have shown that chemotherapy and immunotherapy play different roles in the process of cancer treatment. Traditional neoadjuvant chemotherapy aims to minimize tumor lesions and achieve preoperative downstaging, while neoadjuvant immunotherapy can eliminate micrometastatic tumor lesions by enhancing the anti-tumor immune response (26).

**Figure 2:**
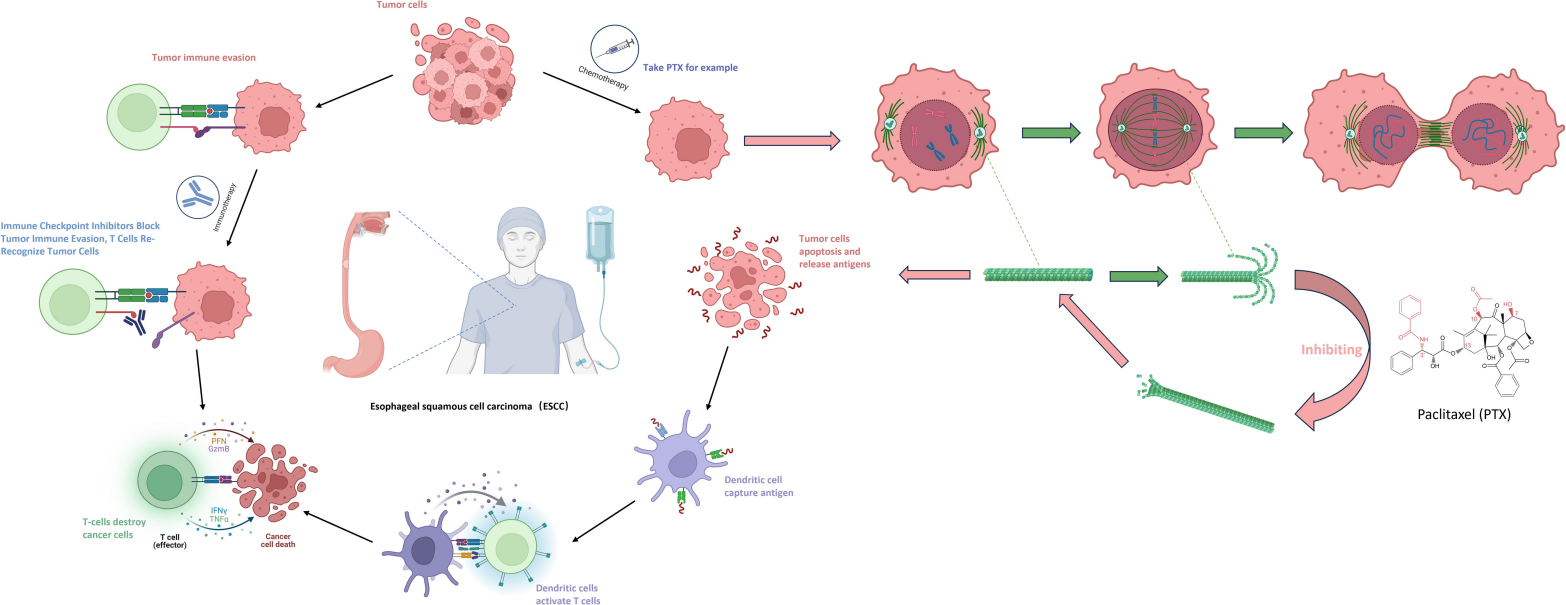
Mechanisms of synergistic effects of immune-combination chemotherapy.

However, current research has put forward new viewpoints regarding the combination treatment of the two. Among ICIs (immune checkpoint inhibitors), PD-L1 monoclonal antibodies can specifically bind to PD-1, blocking the interaction between PD-1 and its ligands, thereby restoring T-cell-mediated immune responses against tumors. By inhibiting the PD-1/PD-L1 signaling pathway, the antitumor activity of T cells is regulated, enhancing the patient’s own immune system response to the tumor and ultimately achieving the goal of killing tumor cells (27). Among chemotherapeutic drugs, taking albumin-bound paclitaxel as an example, the anti-tumor mechanism of taxanes is to induce and promote the polymerization of tubulin into microtubules, while inhibiting the depolymerization of the formed microtubules. This further leads to abnormal arrangement of microtubule bundles, the formation of asters, damage to the DNA of tumor cells, preventing tumor cells from forming normal mitotic spindles during mitosis, thus inhibiting their division and proliferation and causing tumor cell death. Subsequently, the dead tumor cells release tumor antigens, which are captured by dendritic cells and presented to T cells, thereby activating T cells again (28).(**Figure 2**) In addition, some studies have shown that platinum-based chemotherapeutic drugs can cause DNA cross-linking damage in tumor cells, trigger the DNA damage response (DDR), and activate ATM/ATR kinases, thus directly or indirectly promoting the expression of PD-L1 on tumor cells (29). Therefore, the combination of immunotherapy and chemotherapy has a synergistic effect rather than a simple additive one. This also suggests that different chemotherapy regimens combined with immunotherapy may have an impact on the therapeutic effect.

### 2.3 Immunotherapy combined with anti-angiogenic drugs

**Figure 3:**
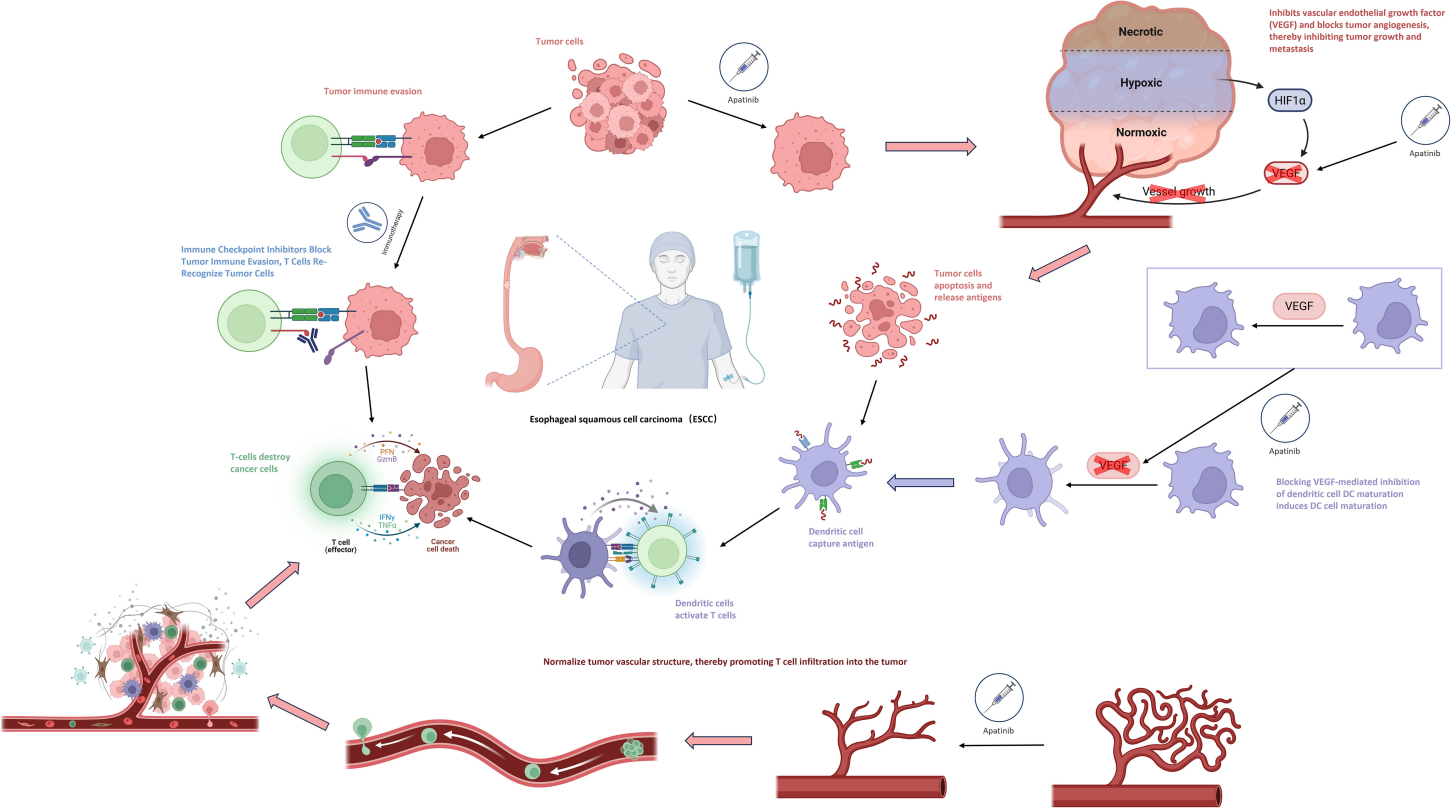
Mechanisms of synergistic action of immunization combined with antiangiogenic drugs.

Immune checkpoint inhibitors (ICIs) and anti-angiogenic drugs may also have a synergistic effect in promoting tumor vascular normalization and stimulating immune activation. Anti-angiogenic drugs (such as apatinib, bevacizumab) can promote the maturation of dendritic cells (DCs) by blocking the VEGF-mediated inhibition of DC maturation, enabling more effective initiation and activation of T cells that bind to tumor antigens. At the same time, they can normalize the structure of tumor blood vessels, thereby facilitating the infiltration of T cells into the tumor. The normalization of tumor blood vessels can promote the aggregation of immune cells and enhance immune function, and the activation of immune cells can, in turn, promote vascular normalization. The combination of the two theoretically can form a positive feedback loop (30, 31)(**Figure 3**).

The combination of immune checkpoint inhibitors (ICIs) and anti-angiogenic drugs has shown certain advantages in the short-term efficacy of neoadjuvant treatment for locally advanced esophageal squamous cell carcinoma (ESCC). However, there are still problems such as a relatively small sample size and the lack of long-term survival outcomes. In recent years, many attempts have been made to use the treatment regimen of immunotherapy combined with anti-angiogenic drugs in other cancers. This combination can significantly improve the objective response rate (ORR) and median progression-free survival (mPFS) of patients, but multiple phase III studies have shown negative overall survival (OS) results (32–34). This poses new challenges to other ongoing trials based on immunotherapeutic drugs, that is, short-term tumor shrinkage does not necessarily bring long-term survival benefits to patients. In this regard, some researchers believe that the combined application of anti-angiogenic drugs and ICIs may have both beneficial and harmful dual effects on tumor immunity. The beneficial effects mainly involve immunostimulatory vascular regulatory effects, which promote the normalization of tumor blood vessels and the formation of an immunostimulatory tumor microenvironment (TME), thus enhancing the anti-tumor immunity. The harmful effects include overpruning of tumor blood vessels, leading to more hypoxia in the TME and further immune suppression, as well as reduced drug biodistribution, which may promote tumor growth and/or metastasis (35).

## 3 Clinical studies on perioperative immunotherapy

### 3.1 Neoadjuvant therapy

#### 3.1.1 Immunotherapy combined with chemotherapy

The KEYSTONE studies represent investigator-initiated prospective clinical trials evaluating pembrolizumab in the perioperative management of Chinese ESCC patients. Among them, KEYSTONE-001—the first global trial to assess pembrolizumab plus paclitaxel/cisplatin as neoadjuvant therapy for locally advanced (AJCC stage III) ESCC—is a single-arm, single-center study enrolling 50 patients. Primary endpoints include major pathological response (MPR) and safety. Interim results presented at the 2021 ESMO OI Congress demonstrated: In 47 evaluable patients, no grade 3 adverse events were reported. Nutritional status improved from baseline, correlated with increased median body weight and Nutritional Risk Index (NRI). Quality-of-life metrics (EORTC QLQC30 functional/symptom scales and EORTC QLQ-OES18 items) showed significant improvements from baseline to post-adjuvant therapy. Efficacy outcomes in 45 evaluable patients included: MPR rate: 73.3%, pCR rate 42.2%, ORR rate 95.6%, DCR rate 100%. With a median follow-up of 23.3 months (95% CI 20.6–24.9; max 34 months), 1-year OS/DFS rates were 95.6%, and 2-year OS/DFS rates reached 90.5%/86.3% (36). KEYSTONE-001’s pembrolizumab-chemotherapy regimen demonstrated unprecedented safety and efficacy profiles, warranting further investigation in randomized controlled trials.

The FRONTiER trial, presented at the 2021 ASCO Annual Meeting, marked Japan’s first exploration of immune checkpoint inhibitors (nivolumab) combined with standard cisplatin + 5-fluorouracil in locally advanced resectable ESCC. Despite enrolling only 13 patients, the regimen demonstrated promising efficacy and tolerability (37). Concurrently, the TD-NICE study presented at ESMO-IO 2021 evaluated tislelizumab plus chemotherapy as neoadjuvant therapy for resectable ESCC. This single-arm phase II trial enrolled 45 treatment-naive patients who received 3 cycles of tislelizumab combined with albumin-bound paclitaxel and carboplatin. Primary endpoints included major pathological response (MPR), with secondary endpoints of pathological complete response (pCR) and overall survival (OS). Safety was assessed via treatment-related adverse events (TRAEs) and postoperative complications. Results demonstrated: In the TD-NICE trial, among 36 (80.0%) operated patients, R0 resection was achieved in 29 (80.5%), with 75% experiencing tumor downstaging. Notably, major pathological response (MPR) and pathological complete response (pCR) rates reached 72.0% and 50.0%, respectively. In the intention-to-treat (ITT) population (n=45), MPR and pCR rates were 57.5% and 40%. Safety outcomes included grade 3–4 treatment-related adverse events (TRAEs) in 42.2% and immune-related AEs (IRAEs) in 22.2% of patients. Postoperative complications occurred in 77.8% of operated patients, yet no treatment-related surgical delays or deaths were reported. No association was observed between genetic mutations and pathological response (38).TD-NICE’s encouraging results validate tislelizumab-chemotherapy as a tolerable, active regimen for resectable ESCC, supporting further investigation.

ChiCTR2000028900, a single-arm, prospective phase II trial, evaluated the efficacy and safety of camrelizumab combined with nab-paclitaxel and carboplatin (2 cycles) as neoadjuvant therapy for resectable ESCC patients (stage II/III). Primary endpoints included treatment-related adverse event (AE) rates and surgical safety, while secondary endpoints comprised major pathological response (MPR), R0 resection rate, objective response rate (ORR), disease control rate (DCR), disease-free survival (DFS), and overall survival (OS). The study enrolled 23 patients, demonstrating acceptable tolerability with no observed surgical delays. Grade 3–4 AEs included neutropenia (9/23, 39.1%) and leukopenia (2/23, 8.7%). Impressively, ORR and DCR reached 90.5% and 100%, respectively. Among 20 operated patients, all achieved R0 resection, with 5 (25%) achieving pathological complete response (pCR) and 10 (50%) demonstrating major pathological response (MPR) (39).

The NICE trial, a single-arm phase II study published in 2022, evaluated the safety and efficacy of camrelizumab combined with chemotherapy as neoadjuvant therapy for locally advanced ESCC. Enrolling 60 patients with resectable disease at more advanced stages (T1b-4a, N2-3 ≥3 lymph nodes, M0/M1 limited to supraclavicular nodes), the trial administered 2 cycles of camrelizumab + nab-paclitaxel + carboplatin. Primary endpoints included pathological complete response (pCR) in the per-protocol population, while secondary endpoints spanned R0 resection rate, disease-free survival (DFS), and overall survival (OS). Safety was assessed in the modified intention-to-treat population receiving ≥1 camrelizumab dose. In the NICE trial, 55 (91.7%) patients completed two cycles of camrelizumab-chemotherapy, with 51 proceeding to surgery. Surgical outcomes included R0 resection in 50/51 (98.0%) patients, pathological complete response (pCR) in 20/51 (39.2%), and primary tumor regression with residual nodal disease (ypT0N+) in 5 (9.8%). Safety data showed any-grade treatment-related adverse events (TRAEs) in 58/60 (96.7%) patients, most commonly leukopenia (86.7%). Grade 3+ adverse events occurred in 34 (56.7%), including one (1.7%) non-treatment-related grade 5 event, with no in-hospital/postoperative deaths within 30/90 days (40) Updated 2023 AATS results revealed 2-year overall survival (OS) and disease-free survival (DFS) rates of 76.9% and 65.2%, respectively (41). Notably, NICE validated camrelizumab-chemotherapy as a regimen with robust anti-tumor activity (pCR 39.2%, R0 98.0%) and manageable toxicity, even in later-stage ESCC. These findings position this combination as a promising neoadjuvant strategy for locally advanced disease, balancing efficacy with safety.

The NIC-ESCC2019 trial, an open-label, multicenter, single-arm phase II study, aimed to evaluate the efficacy and safety of camrelizumab combined with chemotherapy in resectable locally advanced ESCC. Enrolling 56 patients with clinical stage II-IVA disease, the trial administered two cycles of neoadjuvant therapy prior to surgery. Primary endpoints included complete pathological response (CPR) of the primary tumor, while secondary endpoints encompassed objective response rate (ORR) per RECIST v1.1, 2-year progression-free survival (PFS), overall survival (OS), progression-free survival (PFS), and safety during neoadjuvant chemotherapy (NIC) and perioperative periods. In the NIC-ESCC2019 trial, complete pathological response (CPR) of the primary tumor was observed in 35.3% (95% CI, 21.7%-48.9%) of 51 operated patients, with an objective response rate (ORR) of 66.7% (95% CI, 40.0%-70.4%). The safety profile was favorable, with low-grade treatment-related adverse events (TRAEs) predominating: grades 1–2 in 75.0%, grade 3 in 10.7%, and no grades 4–5 events. Notably, no perioperative deaths were reported during the study period (42). This study provided a viable and effective treatment option for esophageal cancer, though long-term survival analysis remains ongoing.

A single-arm, phase II clinical trial evaluated the efficacy and safety of neoadjuvant camrelizumab combined with paclitaxel and nedaplatin in locally advanced ESCC (stages IIa–IIIb). The study featured flexible treatment cycles, allowing patients to elect surgery after receiving at least two cycles of therapy based on their preference. Enrolling 75 patients, the trial administered 2–4 cycles of neoadjuvant therapy followed by surgery. Primary endpoints included pathological complete response (pCR) rate, with secondary endpoints comprising major pathological response (MPR) rate, R0 resection rate, tumor regression, objective response rate (ORR), and disease-free survival (DFS). Results showed that 45 patients (60%) received 2 cycles of neoadjuvant therapy, 18 (24%) received 3 cycles, and 10 (13.3%) received 4 cycles. Among 62 patients (82.7%) who underwent surgery, all achieved R0 resection. Pathological outcomes included a pCR rate of 27.4% (95% CI: 16.9–40.2), MPR rate of 45.2% (95% CI: 33.1–59.2), and ORR of 48.4% (95% CI: 35.5–61.4). Tumor downstaging occurred in 39 (62.9%) patients for T classification and 19 (30.6%) for N classification. Safety data revealed treatment-related adverse events (TRAEs) in 59 patients (78.7%, grades 1–2), with grade 3 TRAEs in 4 (5.3%) and grade 4 TRAEs in 1 (1.3%) (43). The study results demonstrate the feasibility of neoadjuvant camrelizumab combined with paclitaxel and nedaplatin in locally advanced ESCC, with manageable safety profiles across treatment cycles (2–4).

The KEEP-G 03 trial, an open-label, single-arm phase II study, evaluated the efficacy and safety of sintilimab combined with triplet chemotherapy (liposomal paclitaxel, cisplatin, and S-1) for 2 cycles in resectable locally advanced ESCC. Primary endpoints included safety and surgical feasibility, with secondary endpoints such as major pathological response (MPR) rate and R0 resection rate. Results showed that all 30 patients completed two cycles of neoadjuvant therapy and underwent surgery. Grade 3–4 treatment-related adverse events (TRAEs) occurred in 36.7% (11/30), with the most common TRAEs being leukopenia (76.7%), anemia (76.7%), and neutropenia (73.3%). All TRAEs were hematological toxicities, and no surgical delays exceeding 30 days were observed. Pathological outcomes included a major pathological response (MPR) rate of 50.0% (15/30; 95% CI: 33.2–66.9) and a pathological complete response (pCR) rate of 20.0% (6/30; 95% CI: 9.5–37.3) (44). The findings of this study provided the first evidence of the feasibility of sintilimab combined with triplet chemotherapy as a neoadjuvant regimen for resectable ESCC, though further validation with larger sample sizes is warranted.

ChiCTR1900026593 is a single-center, single-arm phase II clinical trial enrolling 47 resectable ESCC patients (stages II–IVA), with 33 (70.2%) classified as clinical stage III. All participants received 2 cycles of neoadjuvant sintilimab combined with liposomal paclitaxel and carboplatin. Primary endpoints included efficacy (pathological complete response [pCR] rate) and safety, while secondary endpoints comprised disease control rate (DCR), disease-free survival (DFS), tumor regression grade (TRG), and overall survival (OS). Results demonstrated that among 45 patients undergoing curative surgery, 44 (97.8%) achieved R0 resection, with 10 (22.2%) achieving pathological complete response (pCR) and 20 (44.4%) demonstrating major pathological response (MPR). In terms of safety, grade 3–4 treatment-related adverse events (TRAEs) included neutropenia (6/47, 12.8%) and leukopenia (8/47, 17.0%), with one case (2.1%) of immune-related encephalitis reported (45). The study demonstrated a relatively high pathological complete response (pCR) rate and manageable safety profile, suggesting that the neoadjuvant regimen of sintilimab combined with liposomal paclitaxel and carboplatin warrants further investigation.

The NCT04460066 trial, the first global multicenter, randomized, double-blind, placebo-controlled phase II study of neoadjuvant immunotherapy plus chemotherapy for locally advanced ESCC, enrolled 64 patients randomized 1:1 to receive either socazolimab combined with nab-paclitaxel and cisplatin (n=32) or placebo plus the same chemotherapy backbone (n=32). Conducted in two stages (phase Ib and II), the trial involved four cycles of therapy followed by surgery. Primary endpoints included major pathological response (MPR), with secondary endpoints comprising pathological complete response (pCR), R0 resection rate, event-free survival (EFS), overall survival (OS), and safety. Results showed that 29 patients (90.6%) in each arm underwent surgery, with R0 resection achieved in 100% of the socazolimab+TP group (n=29) and 98.6% of the placebo+TP group (n=28). MPR rates were 69.0% vs. 62.1% (95% CI: 49.1–84.0% vs. 42.4–78.7%, P=0.509), while pCR rates were 41.4% vs. 27.6% (95% CI: 24.1–60.9% vs. 13.5–47.5%, P=0.311). Notably, the socazolimab+TP group demonstrated significantly higher rates of ypT0 status (37.9% vs. 3.5%, P=0.001) and T downstaging compared to placebo+TP, without increased surgical complications (46). The favorable efficacy and safety profiles demonstrated in NCT04460066 suggest that socazolimab plus nab-paclitaxel/cisplatin (TP) represents a promising neoadjuvant treatment strategy for locally advanced ESCC, warranting further investigation.

ChiCTR1900027160 is a single-arm, single-center phase II clinical trial evaluating the efficacy and safety of toripalimab combined with nab-paclitaxel and S-1 in locally advanced resectable ESCC. Enrolling 60 patients with stage II/III/IV non-metastatic ESCC, the trial’s primary endpoint was major pathological response (MPR), with secondary endpoints including pCR, ORR, DCR, DFS, OS, improvements in dysphagia scores, and daily activity of living (dADL). In this phase II trial, toripalimab-nab-paclitaxel-S-1 achieved 98.2% R0 resection with 49.1% MPR and 29.1% pCR. Treatment improved functional outcomes (dysphagia/dADL) and showed manageable toxicity (18.3% grade ≥3 AEs), with PD-L1-high patients demonstrating superior responses (47). Toripalimab-nab-paclitaxel-S-1 showed promising activity and tolerability in ESCC, as demonstrated by the ChiCTR1900027160 trial.

The ESCORT-NEO trial, China’s first multicenter, randomized, parallel-controlled Phase III study comparing camrelizumab plus chemotherapy versus chemotherapy alone as neoadjuvant therapy for resectable locally advanced esophageal squamous cell carcinoma (ESCC), presented updated results at the 2024 ASCO GI Symposium. The trial enrolled 391 patients across three parallel arms: Arm A (camrelizumab + nab-paclitaxel/S-1), Arm B (camrelizumab + paclitaxel/S-1), and Arm C (paclitaxel/S-1). Results showed that in the intention-to-treat (ITT) population, pCR rates in Arm A (28.0%) and Arm B (15.4%) were significantly higher than those in Arm C (4.7%) (Arm A vs. Arm C: difference: 23.5%, 95% CI: 15.1–32.0, OR: 8.11, 95% CI: 3.28–20.06; P < 0.0001; Arm B vs. Arm C: difference: 10.9%, 95% CI: 3.7–18.1; OR: 3.83, 95% CI: 1.48–9.80; P=0.0034). Major pathologic response (MPR) rates were 59.1%, 36.2%, and 20.9% in Arms A, B, and C, respectively. In the surgery-treated population, R0 resection rates reached 99.1%, 95.7%, and 92.2% for Arms A, B, and C, with postoperative complication incidences of 34.2%, 38.8%, and 32.0%, respectively. During neoadjuvant therapy, grade 3 treatment-related adverse event rates were 34.1%, 29.2%, and 28.8% across Arms A, B, and C (48). The findings of ESCORT-NEO further validate that neoadjuvant immunotherapy plus chemotherapy offers superior efficacy and safety compared to chemotherapy alone. Building on these results, the 2024 CSCO Guidelines for the Diagnosis and Treatment of Esophageal Cancer have listed camrelizumab combined with albumin-bound paclitaxel/paclitaxel and cisplatin as a Grade I expert recommendation for neoadjuvant therapy in resectable locally advanced thoracic ESCC.

#### 3.1.2 Immunotherapy combined with anti-angiogenic drugs

Building on the promising efficacy and safety of PD-1 inhibitors combined with anti-angiogenic drugs in advanced ESCC second-line treatment, the NCT03917966 trial presented at the 2024 ASCO Annual Meeting explored the use of camrelizumab plus apatinib as neoadjuvant therapy for locally advanced resectable ESCC. This open-label, non-randomized phase II clinical trial enrolled 24 patients with cT2-4aN0-3M0 stage ESCC. In this phase II trial, camrelizumab-apatinib achieved ORR/DCR of 50%/95%, with 100% R0 resection and 42.1% MPR in surgical patients. TNM downstaging occurred in 68.4%, supporting further evaluation of this regimen (49).

ChiCTR2200064848 is a prospective single-arm phase II trial evaluating anlotinib combined with penpulimab as neoadjuvant therapy for resectable locally advanced esophageal squamous cell carcinoma (ESCC). Using a Simon two-stage design, the trial enrolled 25 patients in the first stage. If ≥3 patients achieved pathological complete response (pCR), an additional 15 patients would be enrolled in the second stage. The study included 25 locally advanced ESCC patients ineligible for neoadjuvant chemoradiotherapy or chemotherapy, who received 2 cycles of preoperative treatment. In this phase II trial, anlotinib-penpulimab achieved 87.5% R0 resection with 18.8% pCR and 31.3% MPR. TRAEs were manageable (84% grade 1–2, 12% grade 3), supporting further evaluation of this regimen in ESCC (50). These findings demonstrate that neoadjuvant immunotherapy combined with anti-angiogenic agents yields moderate efficacy in ESCC, with pathological response rates lower than those of neoadjuvant chemoradiotherapy or chemo-immunotherapy. However, this regimen offers superior safety profiles, particularly for ESCC patients ineligible for standard neoadjuvant chemoradiotherapy or chemotherapy.

Neoadjuvant immunotherapy combined with anti-angiogenic therapyachieves favorable pathological responses and tumor downstaging in locally advanced resectable ESCC, providing a novel approach for selecting combinatorial neoadjuvant immunotherapy strategies in this population.

The above-mentioned clinical trials are summarized in **Table 1**.

**Table 1:** Clinical research on neoadjuvant immunotherapy for esophageal squamous cell carcinoma (ESCC).

Research name	Research stage	Case number	Research cohort	Patient stage	pCR	MPR	Grade 3-4 AE
Keystone-001	II	49	Pembrolizumab + paclitaxel + cisplatin	III	42.20%	73.30%	0(0/47)
FRONTiER	I	13	Nivolumab+CDDP + 5-FU	I-IVa	/	33.00%	50.0%(6/12)
TD-NICE	II	45	Tislelizumab + Albumin - bound Paclitaxel + Carboplatin	II-IVa	50%	72%	64.4%(29/45)
ChiCTR2000028900	II	23	Camrelizumab + Albumin - bound Paclitaxel + Carboplatin	II-III	25.00%	50.00%	47.8%(11/23)
NICE	II	60	Camrelizumab + Albumin - bound Paclitaxel + Carboplatin	III-IVa	39.20%	68.60%	56.7%(34/60)
NIC-ESCC2019	II	56	Camrelizumab + Albumin - bound Paclitaxel + cisplatin	II-IVa	35.30%	58.80%	10.7%(6/56)
ChiCTR2000033761	II	75	Camrelizumab + Paclitaxel + Nedaplatin	IIa-IIIb	27.40%	45.20%	6.7%(6/75)
NCT03917966	II	24	Camrelizumab + Apatinib	II-IVa	10.50%	42.10%	8.3%(2/24)
KEEP-G 03	II	30	Sintilimab + Paclitaxel Liposome + Cisplatin + S-1	I-IVa	20.00%	50.00%	36.7%(11/30)
ChiCTR1900026593	II	47	Sintilimab + Paclitaxel Liposome + Carboplatin	II-IVa	22.20%	44.40%	29.8%(14/47)
NCT04460066	Ib/II	64	Socazolimab + Albumin-bound Paclitaxel + Cisplatin / Placebo + Albumin-bound Paclitaxel + Cisplatin	II-IVa	41.1% vs. 27.6%	69.0% vs. 62.1%	65.6%(21/32)vs.62.5%(20/32)
ChiCTR1900027160	II	60	Toripalimab + Albumin-bound Paclitaxel + S-1	II-IV	29.09%	49.09%	18.3%(11/60)
ChiCTR2200064848	II	25	Penpulimab + Anlotinib	III-Iva	18.80%	31.30%	12.0%(3/25)
ESCORT-NEO	III	391	Camrelizumab + Albumin-bound Paclitaxel + Cisplatin / Camrelizumab + Paclitaxel + Cisplatin / Paclitaxel + Cisplatin	I-IVa	28.0% vs. 15.4% vs. 4.7%	59.1% vs. 36.2% vs. 20.9%	34.1% vs. 29.2% vs. 28.8%

### 3.2 Postoperative adjuvant therapy

#### 3.2.1 Single-agent immunotherapy

The CheckMate-577 trial, the first global phase III study to demonstrate positive outcomes for adjuvant immunotherapy in esophageal cancer, enrolled 794 patients to evaluate nivolumab versus placebo as adjuvant therapy in individuals with residual pathology following nCRT and complete resection of esophageal or esophagogastric junction cancer. The primary endpoint was DFS, with secondary endpoints including OS and 1-, 2-, and 3-year OS rates. With a median follow-up of 24.4 months, the median DFS was 22.4 months (95% CI: 16.6–34.0) in the nivolumab arm (n=532), compared to 11.0 months (95% CI: 8.3–14.3) in the placebo arm (n=262), representing a significant HR of 0.69 (96.4% CI: 0.56–0.86, P<0.001) (51). CheckMate-577 confirmed nivolumab’s role in adjuvant esophageal cancer, with durable improvements in DFS,DMFS that align with its mechanism of overcoming minimal residual disease (52). Building on CheckMate-577’s robust efficacy, nivolumab was incorporated into authoritative esophageal cancer guidelines (CSCO, NCCN) in 2022, establishing it as the first recommended adjuvant immunotherapy. While sparking interest in ESCC adjuvant care, CheckMate-577 has limitations: it enrolled only patients failing to achieve pCR after nCRT, which is not globally standard. Whether nivolumab’s benefit extends to alternative preoperative strategies remains unclear. Ongoing studies evaluate ICIs in esophageal cancer adjuvant settings across diverse neoadjuvant paradigms.

The HCHTOG2203 trial is a Chinese multicenter, two-arm, open-label Phase III randomized controlled trial enrolling patients with histologically confirmed residual disease after neoadjuvant chemotherapy for thoracic ESCC or incidentally detected pathological lymph node metastasis in clinical T1-2N0 ESCC following upfront surgery. Eligible patients are randomized 2:1 to adjuvant sintilimab or observation. Primary endpoint is DFS, with secondary endpoints including OS, AE, quality of life (QOL) assessment, and nutritional risk screening (NRS) (53). The study aims to demonstrate superior DFS with adjuvant sintilimab compared to control in locally advanced ESCC not achieving pCR after neoadjuvant therapy. Currently ongoing, HCHTOG2203 seeks to establish safe and effective adjuvant options for ESCC patients receiving preoperative chemotherapy.

KEYSTONE-002 is a multicenter, prospective, randomized controlled phase III clinical study. This study consists of two parts. First, 342 patients with locally advanced resectable esophageal squamous cell carcinoma (ESCC) (staging: cT1N2M0 or cT2-3N0-2M0 (stage II/III, high-risk lesions in T2N0M0)) were enrolled and randomly assigned to the experimental group (pembrolizumab combined with neoCT, n = 228) or the control group (neoCRT, n = 114) at a ratio of 2:1 to receive neoadjuvant treatment. Surgical resection was performed 4–6 weeks later. Patients in the experimental group will also receive pembrolizumab alone as adjuvant treatment after surgery until 1 year, or until radiologically confirmed progression of disease (PD) or other situations requiring early termination are observed. The primary endpoint is event-free survival (EFS). The secondary endpoints include overall survival (OS) and disease-free survival (DFS) at 1 year, 3 years and 5 years, short-term outcomes, and quality of life (54). KEYSTONE-002 was initiated in December 2021 and is currently still ongoing. Compared with other studies, KEYSTONE-002 has a larger and more sufficient sample size. We look forward to the announcement of the study results, which may answer the question of whether adjuvant pembrolizumab after neoadjuvant immunotherapy combined with chemotherapy is effective in the future. It is of great significance to open up a brand-new neoadjuvant treatment mode for Chinese patients with esophageal squamous cell carcinoma.

#### 3.2.2 Immunotherapy combined with chemotherapy

AIRES is a multicenter phase III clinical trial led by Chinese researchers, aiming to evaluate the efficacy and safety of adjuvant chemotherapy combined with tislelizumab versus tislelizumab alone in the treatment of patients with high-risk (y)pN+ esophageal squamous cell carcinoma (ESCC) after radical resection. The key inclusion criteria include (y)pN+ after neoadjuvant chemoradiotherapy or chemotherapy plus surgery or pre-operative R0 resection. Eligible patients (n = 220) will be randomly assigned (1:1) to receive adjuvant chemotherapy (once every 3 weeks for two cycles), followed by tislelizumab 200 mg administered intravenously every 3 weeks for 1 year, or tislelizumab 200 mg administered intravenously every 3 weeks for 1 year (55). This study is currently ongoing, and its findings will also influence the treatment choices of adjuvant immunotherapy after perioperative chemotherapy for the Asian population.

#### 3.2.3 Immunotherapy combined with anti-angiogenic drugs

The ALTER-E005 study explored the efficacy and safety of adjuvant immunotherapy combined with anlotinib for the treatment of esophageal squamous cell carcinoma (ESCC) after direct surgery. This is a single-arm, multicenter Phase II clinical study. A total of 12 patients with ESCC who had undergone radical resection and were diagnosed with T1-2N1-3M0 or T3-4NanyM0 were enrolled and received adjuvant treatment with anlotinib combined with bemosiranib. As of August 2023, with a median follow-up of 5.1 months, the primary endpoint, disease-free survival (DFS), had not been reached. 25% of the patients experienced grade 3 treatment-related adverse events (TRAEs), and there were no TRAEs of grade 4 or higher (56). The study indicates that the combined adjuvant treatment of anlotinib and bemosiranib for ESCC patients has manageable safety. However, due to the small sample size and short follow-up time of this study, the efficacy remains uncertain.

The above-mentioned clinical trials are summarized in **Table 2**.

**Table 2:** Ongoing clinical studies for ESCC.

Study	Phase	Study design	Sample size	Study arms / intervention	Patient population	Primary endpoint	Secondary endpoints	Start date	Estimated completion date	Status
HCHTOG2203	III	Two-arm	219	Sintilimab vs. Observation	ESCC patients who did not achieve pCR after surgery after nCT, or patients with clinical T1-2N0 with incidental pathologic lymph node metastases after surgery	DFS	OS, AE, QOL, NRS	2022/8/1	2025/8/31	Recruiting
KEYSTONE-002	III	2-stage, Single-arm	228	Pembrolizumab	ESCC patients with surgery after nCIT	EFS	1,3,5-year OS and DFS	2021/12/1	2028/5/1	Recruiting
AIRES	III	Two-arm	220	Tislelizumab + Chemotherapy vs. Tislelizumab	ESCC patients with high risk of postoperative recurrence type	DFS	OS, AE, QOL	2021/5/1	2028/4/30	Recruiting
ALTER-E005	II	Single-arm	30	Anlotinib + TQB2450	ESCC patients with T1-2N1-3M0 or T3-4NanyM0 after surgery	DFS	1,3-year DFS rate; 1,3-year OS rate	2022/6/2	2026/11/1	Recruiting

## 4 Immune-related adverse reactions

The emergence of immune checkpoint inhibitors (ICIs) has brought hope to cancer patients. However, cancer immunotherapy is not a panacea. A series of new immune-related adverse events (IRAEs) have emerged during the treatment process, and these adverse reactions are usually significantly different from the traditional chemotherapy-related toxicities. Especially when immunotherapeutic drugs are combined with chemotherapeutic drugs for treatment, it greatly increases the incidence of adverse events. Multiple meta-analyses have shown that the incidence of adverse event toxicities in the combination of anti-PD-1, anti-PD-L1 and anti-CTLA-4 is significantly higher than that in anti-PD-1 or anti-PD-L1 monotherapy (57–60). This may be caused by the greater impact of the combination therapy on each step in the immune cycle process (24). Common immune-related adverse events (irAEs) are shown in **Table 3**. In addition, there are other rarer irAEs, such as cardiovascular toxicity, neurological toxicity, renal toxicity, hematological toxicity, and ocular toxicity, etc. (61, 62). The incidence of fatal ICI-related adverse reactions is approximately 0.3% to 1.3%. Although the incidence is relatively low, it often leads to devastating clinical consequences. For example, cardiovascular complications caused by ICI treatment have a high mortality rate, and patients often die due to refractory arrhythmia or cardiogenic shock.

**Table 3:** Common immune-related adverse events (IRAE).

irAEs	Incidence rate	Clinical manifestations	Highly prevalent types of ICIs
Cutaneous toxicity	70%	Psoriasis, pruritus, macular rash and eczematous reactions	CTLA-4、PD-1/PD-L1
Toxicity of the digestive system	5%~30%	Diarrhea, colitis, hepatitis, gastritis and enterocolitis	CTLA-4
Musculoskeletal toxicity	10%	Arthralgia and myalgia	CTLA-4、PD-1/PD-L1
Pulmonary toxicity	0~10%	Pneumonia	PD1/PD-L1
Toxicity of the endocrine system	0~10%	Hypothyroidism, thyrotoxicosis, hypophysitis, adrenocortical insufficiency, diabetes mellitus	PD-1/PD-L1

The fatal toxicities of ICIs often occur in the early stage of the treatment process and develop rapidly, especially in patients receiving combined drug therapy, with a higher frequency of occurrence. The spectrum of fatal IRAEs varies greatly among different treatment regimens. A meta-analysis showed that colitis was the most common cause of irAE-related death among patients receiving anti-CTLA-4 antibodies [135 out of 193 deaths (70%)], while the deaths of patients receiving anti-PD-1 or anti-PD-L1 antibodies were mainly attributed to pneumonia [115 out of 333 cases (35%)], hepatitis [75 out of 333 cases (22%)], and neurotoxic effects [50 out of 333 cases (15%)]. Among patients receiving combination therapy, ICI-related deaths were mainly attributed to colitis [32 out of 87 cases (37%)] or myocarditis [22 out of 87 cases (25%)] (63).

## 5 The future development directions of immunotherapy for esophageal squamous cell carcinoma

### 5.1 The dose intensity of neoadjuvant immunotherapy combined with chemotherapy

It is worth noting that in the clinical studies of neoadjuvant immunotherapy combined with chemotherapy, the pathological complete response (pCR) rate generally remains at around 20%. However, the pCR rate in the TD-NICE study is as high as 50%, especially in the NICE study. Even though the included patients had relatively advanced pathological stages, the pCR rate could still reach 39.2%. This may be because the chemotherapy doses in these two studies were higher than those in other clinical studies. In the NICE study, the dose of albumin-bound paclitaxel was 100 mg/m², administered on days 1, 8, and 15. The total dose for 2 cycles reached 600 mg/m². In the TD-NICE study, the total dose of albumin-bound paclitaxel for 3 cycles reached 780 mg/m², along with carboplatin (area under the curve on day 1 = 5).

A retrospective study analyzed the differences in 122 patients with resectable esophageal squamous cell carcinoma (ESCC) after neoadjuvant immunotherapy combined with different chemotherapy dose intensities. All patients received at least 2 cycles of neoadjuvant tislelizumab combined with chemotherapy. The primary endpoints were pCR and major pathological response (MPR), and the secondary endpoints were the objective response rate (ORR), disease control rate (DCR), and disease-free survival (DFS).

The results showed that 99 patients underwent surgery. According to the chemotherapy dose intensity, the patients were divided into three cohorts: Cohort 1 (<80% dose intensity), Cohort 2 (80-90% dose intensity), and Cohort 3 (90-100% dose intensity). The average pCR rate was 22.22%. In Cohort 1, 16% of the patients achieved pCR; in Cohort 2, 17.65% of the patients achieved pCR; and in Cohort 3, 30.00% of the patients achieved pCR. The number of patients who achieved MPR in the three cohorts were 9 (36.00%), 18 (52.94%), and 22 (55.00%) respectively.

In both univariate and multivariate analyses, the dose intensity was significantly correlated with the MPR of patients who underwent esophagectomy (p = 0.048). In terms of survival, the median follow-up time after esophagectomy was 13.76 months. Compared with Cohort 1, Cohorts 2 and 3 had better DFS (p = 0.056). Moreover, the prognosis of patients who achieved MPR was better than that of those who did not achieve MPR (P=0.005) (64). At the same time, the impact of increasing the chemotherapy dose on safety is also within an acceptable range. Therefore, appropriately increasing the chemotherapy dose within the controllable safety range may lead to more significant therapeutic effects.

### 5.2 Neoadjuvant treatment with different combinations of immune checkpoint inhibitors and chemotherapy for resectable esophageal squamous cell carcinoma

PD-1/PD-L1 immune checkpoint inhibitors all act on the PD-1/PD-L1 signaling pathway, blocking the binding between tumor cells and T cells, countering the immune escape of tumor cells, restoring and enhancing the function of the body’s immune system, and thus exerting anti-tumor activity (15, 16). The difference between them is that PD-1 inhibitors act on the immune cells (T cells) in the tumor microenvironment. They remove the Fc segment on T cells, avoiding the reduction in the number of T cells, and thus play a role. While PD-L1 acts directly on tumor cells. Therefore, it is necessary to optimize the Fc segment of T cells and retain the function of Fc to further enhance the anti-tumor effect. In addition, different PD-1 or PD-L1 inhibitors have different degrees of modification of the Fc segment (65, 66). These differences also lead to variations in the potential anti-tumor activity and safety of PD-1/PD-L1 immune checkpoint inhibitors. A meta-analysis compared the heterogeneity among different ICI inhibitors. The results showed that neoadjuvant treatment with pembrolizumab and tislelizumab showed higher major pathological response (MPR) rates (pembrolizumab: 72.4%, tislelizumab: 72.2%) and pathological complete response (PCR) rates (pembrolizumab: 41.5%, tislelizumab: 50.0%), while the neoadjuvant treatment based on toripalimab and sintilimab had relatively lower MPR rates (toripalimab: 50.0%, sintilimab: 48.5%) and PCR rates (toripalimab: 18.0%, sintilimab: 26.5%). In terms of safety, the overall incidences of treatment-related adverse events (TRAEs) in the neoadjuvant treatment subgroups of camrelizumab, sintilimab, tislelizumab, and toripalimab were comparable (P=0.30), but the pooled incidence of serious adverse events (SAEs) in the neoadjuvant treatment with pembrolizumab seemed to be lower than that of other ICIs (P=0.01) (67). Therefore, it can be considered that different PD-1/PD-L1 inhibitors may lead to differences in neoadjuvant efficacy and safety. However, due to the different stages of patients included in each study in the meta-analysis and the different chemotherapy regimens, it is still not conclusive at present. In the future, more head-to-head studies of different types of ICIs are needed for verification.

### 5.3 The combination methods of various modalities in neoadjuvant immunotherapy

Recently, the long-term follow-up data of the SCALE-1 study, which was successively reported at the 2021 CSCO Congress and the 2022 ASCO Congress, have also been released. This study explored the efficacy and safety of short-course neoadjuvant radiotherapy combined with chemotherapy and toripalimab in the treatment of locally advanced esophageal squamous cell carcinoma (ESCC). The multi-modal combination approach of the SCALE-1 study is different from the traditional neoadjuvant concurrent chemoradiotherapy with immunotherapy, mainly in that the short-course neoadjuvant radiotherapy is arranged between the doses of neoadjuvant immunochemotherapy. That is, on day 1 and day 22, neoadjuvant treatment with toripalimab combined with chemotherapy is used, and neoadjuvant radiotherapy is administered sequentially from day 3 to day 8. Up to now, the 2-year progression-free survival (PFS) rate of this study is 63.8%, and the 2-year overall survival (OS) rate is 78% (21). The good efficacy and innovation of the SCALE-1 study have provided more inspiration for researchers. In multi-modal immunotherapy combinations, adjusting the combination sequence of various modalities to achieve better efficacy and safety has also become a research hotspot in the neoadjuvant treatment of esophageal cancer. In terms of neoadjuvant immunotherapy combined with chemotherapy, some studies have shown that the sequence of preoperative immunotherapy and chemotherapy may also affect the therapeutic effect.

The NCT03985670 study is a single-center, randomized, open-label Phase II clinical study. It enrolled 30 patients with locally advanced resectable ESCC (T3, T4 or positive lymph nodes) and randomly divided them into two groups: receiving chemotherapy on day 1 and immunotherapy on day 3 (experimental group) or receiving chemotherapy and immunotherapy on day 1 (control group). The specific treatment regimen is toripalimab combined with paclitaxel and cisplatin (2 cycles). The primary endpoint is the pathological complete response (pCR) rate, and the secondary endpoints are safety and disease-free survival (DFS) rate.

The results showed that among the 30 patients who completed at least one cycle of neoadjuvant treatment, 11 and 13 patients in the experimental group and the control group, respectively, underwent surgery and all had R0 resection. In the experimental group, 4 patients (36%) achieved pCR, and in the control group, 1 patient (7%) achieved pCR, with no significant statistical difference (P=0.079) (68) However, considering the small sample size of the NCT03985670 study, and the fact that the patient who achieved pCR in the control group was the only one with high PD-L1 expression among all the patients whose PD-L1 expression was detected in this study. Therefore, it can still be considered that in the process of neoadjuvant toripalimab combined with chemotherapy for the treatment of locally advanced resectable esophageal squamous cell carcinoma, delaying toripalimab to the 3rd day after chemotherapy may result in a higher pCR rate compared to using it on the same day.

### 5.4 Acquired resistance during neoadjuvant treatment in patients with esophageal squamous cell carcinoma

The therapeutic function of PD-1/PD-L1 monoclonal antibodies is based on enhancing the pre-existing CD8+ T cells. However, patients with esophageal squamous cell carcinoma (ESCC) may have primary and acquired resistance to PD-1/PD-L1 immunotherapy. Patients with primary resistance often show signs at the initial stage of treatment, and at this time, doctors can adjust the treatment according to the patient’s condition. But acquired resistance is often manifested as a significant shrinkage of the tumor at the initial stage of treatment. However, with the deepening of treatment and the increase in the number of treatment cycles, the disease shows stable disease (SD) or progressive disease (PD), thus missing the opportunity for surgery. Therefore, it is necessary to comprehensively understand the mechanism of acquired resistance of tumors and explore methods to overcome acquired resistance.

Studies have shown that inactivating mutations of JAK1/2, methylation of the PD-L1 promoter, and expression of c-Myc are all considered to be the causes of acquired resistance to immune modulators due to the downregulation of PD-L1. Acquired resistance can also be secondary to the downregulation of B2M, leading to a decrease in the expression of major histocompatibility complex class I (MHC-I) and subsequent escape from CD8+ T cells (69). In addition, some studies have shown that acquired resistance is related to T cell exhaustion, because T cell exhaustion is usually driven by high levels of antigens that persist when the host immune response fails to effectively clear them. These T cells have unique transcriptional and epigenetic characteristics, leading to the overexpression of several inhibitory receptors, changes in metabolic adaptation, and dysregulation of cytokine signaling pathways. Among these overexpressed inhibitory receptors is PD-1, which leads to the occurrence of acquired resistance (70–72).

In order to overcome the acquired resistance of immunotherapy, exploring new drug combinations based on the resistance mechanism or developing new ICI drugs is the fundamental solution. In addition, selecting the most appropriate number of neoadjuvant treatment cycles according to the clinical characteristics of patients can also effectively reduce the incidence of acquired resistance. If surgery can be performed before the occurrence of resistance in ESCC patients, it can significantly improve the preoperative objective response rate (ORR) and R0 resection rate of patients, and reduce the occurrence of adverse events. Currently, there is still a controversy regarding the number of neoadjuvant treatment cycles for ESCC patients in clinical practice. ChiCTR2000033761 only reported that the safety of the regimen of camrelizumab combined with paclitaxel and nedaplatin was controllable when used for 2 to 4 neoadjuvant cycles before surgery, but it did not explain whether there were differences in efficacy among different numbers of cycles.

In 2021, a multi-center, Phase II clinical study was carried out in Japan to compare the efficacy and safety of 2 cycles and 3 cycles of neoadjuvant chemotherapy for the treatment of locally advanced ESCC. The results showed that the surgical completion rate and toxicity were similar between the two groups. The ORR of the 3-cycle group was significantly better (42.9% vs. 65.2%, P=0.0027), and the pCR rate was also relatively higher, but there was no significant statistical difference (9.1% vs. 15.3%, P=0.212) (73). A meta-analysis included 15 studies with a total of 452 resectable ESCC patients to compare the efficacy and safety of 2 cycles and > 2 cycles of neoadjuvant treatment. The results showed that the pooled major pathological response (MPR) rate and pathological complete response (PCR) rate of the two groups were 58.3% and 32.9% respectively, the pooled incidences of treatment-related adverse events (TRAEs) and serious adverse events (SAEs) were 91.6% and 19.4% respectively, and the pooled R0 resection rate was 92.8%. Compared with 2 cycles of neoadjuvant treatment, patients who received > 2 cycles of neoadjuvant treatment had higher MPR rates (57.3% vs. 61.1%) and PCR rates (30.6% vs. 37.9%), and higher incidences of TRAEs (89.2% vs. 98.9%), but there was still no significant difference (P > 0.05), and 2 cycles of neoadjuvant treatment showed a higher R0 resection rate (R0 resection rate: 96.0% vs. 87.8%, P=0.02) (67). In addition, in a real-world analysis of neoadjuvant immunotherapy combined with chemotherapy for non-small cell lung cancer, the results showed that extending the neoadjuvant treatment to 3–4 cycles may improve the safety of surgery and reduce the incidence of postoperative complications, but the MPR rate may not increase significantly (57.3% vs. 57.4%, P=0.529) (74). The increase in the number of treatment cycles has not achieved the expected effects in terms of efficacy and safety. In the future, more trials related to the number of treatment cycles of neoadjuvant treatment for locally advanced ESCC are still needed for exploration.

### 5.5 The timing of surgery after neoadjuvant immunotherapy

Currently, it is generally recommended clinically that patients with resectable esophageal squamous cell carcinoma (ESCC) undergo surgery 4 to 6 weeks after neoadjuvant chemoradiotherapy (nCRT), but the optimal timing of surgery after neoadjuvant immunotherapy has not been clearly defined.

The SCALE-1 study also reported that 8 patients who underwent surgery within 8 weeks after completing preoperative treatment experienced perioperative complications. After extending this interval to more than 8 weeks, no postoperative complications were found, and a trend of weight gain was observed. This indicates that a longer interval, on the premise of ensuring that it does not increase the complexity of the surgery, may also reduce the incidence of acute toxicity related to neoadjuvant treatment and surgery in patients. In addition, no progression was observed in the intention-to-treat (ITT) population, suggesting that a longer interval will not impair the treatment response.

A retrospective study explored whether the time to surgery (TTS) (> 6 weeks) would affect the outcomes. It included 95 patients with locally advanced ESCC who underwent esophagectomy after neoadjuvant immunotherapy combined with chemotherapy. The primary endpoints were pathological complete response (pCR) and disease-free survival (DFS). The results showed that the pCR rate in the standard group was 23.08% (12/52), and that in the extended group was 16.28% (7/43) (P=0.41). Multivariate regression analysis further indicated that TTS was not an independent factor for predicting pCR (P=0.41). The median follow-up time in the standard TTS group was 10.5 months, and that in the extended TTS group was 11.2 months. A total of 5 recurrences occurred, 2 in the standard TTS group and 3 in the extended TTS group, and there was no significant difference in DFS (P=0.60). Moreover, the complications and major complications in the two groups were similar. Therefore, it is believed that the TTS after neoadjuvant immunotherapy combined with chemotherapy is not important for pathological response, disease-free survival rate, and short-term postoperative outcomes (75). In another retrospective study, the outcomes of patients with locally advanced ESCC who had a time interval between surgery after neoadjuvant immunotherapy combined with chemotherapy of ≤ 8 weeks and > 8 weeks were analyzed. The primary endpoints were DFS and overall survival (OS), and the secondary endpoints were pathological response, surgical outcomes, and postoperative complications. The results showed that there were 44 patients in the ≤ 8 weeks group (n = 44) and 36 patients in the > 8 weeks group (n = 36). The major pathological response (MPR) rates were 25.0% in the ≤ 8 weeks group and 27.8% in the > 8 weeks group (P=0.779). The pCR rates were 11.4% in the ≤ 8 weeks group and 16.7% in the > 8 weeks group (P=0.493). The incidences of postoperative complications were 27.3% in the ≤ 8 weeks group and 19.4% in the > 8 weeks group (P=0.413). The median DFS had not been reached in both groups (hazard ratio [HR]: 3.153, 95% confidence interval [CI] 1.383-6.851, P=0.004). The median OS had not been reached in the ≤ 8 weeks group (HR: 3.703, 95% CI 1.584-8.657; P=0.0012), and it was 31.6 months (95% CI 21.1-42.1) in the > 8 weeks group. In the multivariate analysis, poorer DFS and OS were observed in patients with an interval of > 8 weeks (HR: 2.992, 95% CI 1.306-6.851; HR 3.478, 95% CI 1.481-8.170) (76). Therefore, the choice of the timing of surgery after neoadjuvant immunotherapy combined with chemotherapy is still controversial.

Theoretically, the role of immune checkpoint inhibitors is to release the suppression of T cells and activate the immune system to attack tumors. This process requires time. The initiation, expansion, and eventual manifestation of significant tumor regression or pathological response (such as pCR/MPR) are usually not immediate. Clinically and radiologically significant responses may not appear until several weeks or even months after treatment. Premature surgery may interrupt an ongoing effective immune response and miss the opportunity to achieve deeper pathological remission. However, considering that an excessively long interval could lead to the risk of disease progression, we believe that 6 to 8 weeks after neoadjuvant immunotherapy may be the optimal surgical window. Further prospective clinical studies are still needed in the future to verify this.

### 5.6 Limitations of adjuvant immunotherapy

Currently, in clinical practice, patients who have not achieved R0 resection, pCR, or who are found to have lymph node metastasis intraoperatively and are at high risk of recurrence usually undergo adjuvant immunotherapy, which is often initiated within 3 months after surgery; otherwise, the therapeutic efficacy may be reduced, and the risk of recurrence may increase. However, in recent years, there has been ongoing debate regarding the optimal population for adjuvant immunotherapy after esophageal cancer surgery, the best treatment regimen, and even whether adjuvant immunotherapy is necessary at all after surgery.There are differences in the mechanisms of action between adjuvant immunotherapy and neoadjuvant immunotherapy: Neoadjuvant therapy focuses on tumor debulking, so immunotherapy primarily works by reducing T cell-mediated immunosuppression. In adjuvant therapy, after tumor resection, the goal of immunotherapy is to eliminate potential micrometastases that may exist but are undetectable by standard diagnostic tests. Therefore, its mechanism is more likely to involve activating T lymphocytes to clear micrometastases (77). However, after surgical resection, the release of antigens is reduced, and the immune system lacks continuous stimulation, making it difficult to elicit a robust T-cell response. Moreover, lymphadenectomy during surgery can damage the lymphatic system surrounding the tumor bed, further weakening the efficiency of immune cell migration and activation. Therefore, the application of immunotherapy in the neoadjuvant setting is likely to offer more clinical benefits. This conclusion has been validated in mouse animal models, clinical studies, and large-scale informatics studies (78–80).

Given this, some patients may opt for more aggressive adjuvant treatment regimens after surgery, such as immunotherapy combined with chemotherapy or chemoradiotherapy. The overall mechanism of action of adjuvant immunotherapy combined with chemotherapy or radiotherapy includes the unique and crucial effects brought about by chemotherapy or radiotherapy, especially the induction of immunogenic cell death, alteration of the tumor microenvironment, and massive release of antigens. These mechanisms synergize with immunotherapy, significantly differing from the mode of action of immunotherapy alone. Therefore, theoretically, the combination of immunotherapy with chemotherapy or radiotherapy should provide better tumor-killing effects. However, there is currently a lack of research results comparing the long-term survival outcomes of these two approaches.

In addition, the issue of acquired resistance is also evident in adjuvant immunotherapy after surgery. Residual micrometastases after surgery are often accompanied by fibrotic stromal proliferation and high expression of TGF-β, creating a suppressive environment that hinders T-cell infiltration. A recent study retrospectively analyzed data from seven years across eight centers in China, involving a total of 1,428 patients with locally advanced ESCC. The results indicated that whether patients received adjuvant immunotherapy after surgery did not affect OS and DFS (P=0.35) (81). Therefore, adjuvant immunotherapy currently faces many limitations and challenges. Relevant prospective Phase III clinical studies are ongoing (**Table 2**), and we look forward to the publication of the study results, which will provide more theoretical basis for adjuvant immunotherapy.

On the other hand, a real-world analysis of adjuvant treatment after esophageal cancer surgery was conducted in the form of a retrospective cohort study. The immunotherapy cohort was evaluated in terms of treatment completion, adverse reactions, and disease progression, with a focus on patients who underwent surgery in 2021 and their eligibility for receiving nivolumab. The results showed that 39 patients received immunotherapy, while 137 patients did not receive immunotherapy. In the logistic regression analysis, after adjusting for age and receipt of adjuvant chemoradiotherapy, no statistically significant effect of immunotherapy on the 1-year overall survival rate was found. Among the 39 patients who received immunotherapy, only 7 patients successfully completed the treatment (18%), and most of the patients failed due to disease progression or side effects. Among the 39 patients who received immunotherapy, 19 received nivolumab, 17 received pembrolizumab, 11 received trastuzumab, and 2 received ipilimumab. Among the 17 patients who were eligible for nivolumab treatment, 13 patients received nivolumab treatment (76.4%), and 3 patients completed the entire course of treatment (82). Therefore, although adjuvant immunotherapy holds promise for improving the survival of patients with esophageal cancer, the practice in real life differs greatly from that in clinical trials, and most patients are unable to complete the immunotherapy regimen. This will also be one of the challenges faced by adjuvant treatment after esophageal cancer surgery in the future.

### 5.7 The germination of organ preservation for esophageal cancer

Currently, comprehensive treatment mainly based on surgery is the main treatment strategy for patients with early-stage and locally advanced resectable esophageal cancer. However, esophageal cancer surgery has a relatively large trauma and has a great impact on the quality of life of patients. In addition, for locally advanced resectable esophageal cancer, although neoadjuvant chemoradiotherapy is one of the current standard treatment regimens, there has always been a controversy about whether surgical resection is still necessary after achieving a clinical complete response (cCR) following neoadjuvant chemoradiotherapy. In recent years, with the publication of the results of the Dutch pre-SANO trial, “organ preservation” treatment for esophageal cancer has become a hot topic worldwide. The pre-SANO study explored methods for evaluating the clinical response to detect local residual lesions, including endoscopic ultrasound, bite biopsy, and fine-needle aspiration of suspicious lymph nodes, after neoadjuvant chemoradiotherapy for esophageal cancer. PET-CT was used to detect tumor recurrence and metastasis. It preliminarily established the feasibility of the “wait and see” strategy for organ preservation in esophageal cancer and provided a reference for the subsequent phase 3 randomized controlled SANO trial (83). The SANO study divided patients who achieved cCR after neoadjuvant chemoradiotherapy into two groups. One group received SS (standard surgery), and the other group received AS (active surveillance). The primary endpoint of the study was overall survival (OS). The preliminary results of the SANO study were announced at the ESMO conference in 2023. There were 198 patients in the AS group (active surveillance group) and 111 patients in the SS group (standard surgery group). The results showed that 83 patients in the AS group underwent surgery after tumor recurrence, with a median time to surgery of 5.9 months. The complications of the surgical patients in the AS group and the SS group were similar. 35% of the patients in the AS group had a durable response, and 48% of the patients had recurrence. At least 35% of the patients avoided unnecessary surgery due to AS. The 2-year follow-up showed that the primary endpoint was non-inferior, and there were no statistically significant differences in OS and disease-free survival (DFS) (84). Overall, the SANO study provides a key reference for the future organ preservation treatment strategy of esophageal cancer. After that, the pre-SINO study led by Chinese researchers explored for the first time the correlation between changes in circulating tumor DNA (ctDNA) combined with clinical modular examination and pathological outcomes after neoadjuvant chemoradiotherapy (nCRT) in esophageal squamous cell carcinoma (ESCC). ctDNA, detected through tumor DNA fragments in the blood, has high sensitivity and specificity, which can increase accuracy and reduce the false negative rate of cCR patients. The pre-SINO study reported the latest research results at the 2022 ASCO conference. The false negative rate of ctDNA liquid biopsy was only 5%, which was better than the 10% false negative rate in the SANO study (85, 86). It effectively reduced the incidence of clinical misassessment of non-pCR patients, especially bringing a cure opportunity for patients who may miss the surgery in the future due to misassessment as cCR. It suggests that ctDNA liquid biopsy may become an effective active monitoring strategy for the management of ESCC in the future. The treatment regimens of the SANO and SINO series of studies are both concurrent chemoradiotherapy (CROSS regimen). In the context of the immunotherapy era, the combination of immunotherapy and concurrent chemoradiotherapy will enable more patients to achieve a clinical complete response. Theoretically, these patients may not need to undergo surgery and can preserve the esophageal organ. Therefore, the combination of immunotherapy with chemoradiotherapy or the combination of immunotherapy with chemotherapy as a treatment regimen for organ preservation in ESCC patients will be one of the future research focuses. The IKF-t057/PRESTO announced by ASCO-GI is a single-arm phase II study design of the organ preservation strategy for esophageal adenocarcinoma using the combination of immunotherapy with chemoradiotherapy. The study plans to enroll 32 patients with resectable T1-2N0M0 esophageal adenocarcinoma (including adenocarcinoma of the gastroesophageal junction). The enrolled patients will receive durvalumab (once every 4 weeks) and 2 cycles of FLOT (once every 2 weeks) induction therapy simultaneously, followed by 3 cycles of mFOLFOX (once every 2 weeks) and concurrent radiotherapy. The patients will be evaluated 8 weeks after treatment. Patients with confirmed local tumor residue will undergo surgical resection, and patients who achieve cCR will enter the maintenance treatment stage, continuing to receive single-agent durvalumab treatment for up to 12 cycles, and will be regularly examined and re-evaluated. The primary endpoint is the cCR rate/pCR rate at re-evaluation. If the cCR rate/pCR rate ≥ 75%, the treatment regimen will be further studied, while if it is < 55%, no further study is required (87). The first patient was enrolled in August 2023, and the recruitment is still ongoing. It is expected to be completed in August 2024, and we are looking forward to the announcement of the results.

## 6 Conclusion

The excellent efficacy and good safety brought by immunotherapy to patients have promoted its gradual transition from the first- and second-line treatment regimens for advanced or metastatic cases to the perioperative treatment strategies for resectable locally advanced esophageal squamous cell carcinoma (ESCC). Although currently, immunotherapy still lacks large-sample clinical trials to verify its comprehensiveness and stability, the substantial progress of perioperative immunotherapy for ESCC is obvious. Under the current situation, multi-center and multi-cohort randomized controlled trial (RCT) studies of immunotherapy for ESCC are currently underway. The application of immunotherapy in organ preservation still needs to be observed and explored. It is expected that more excellent research results will provide more evidence for immunotherapy. In the near future, perioperative immunotherapy will become a key component of the comprehensive treatment for resectable locally advanced ESCC. In addition, determining the best drug or the most effective treatment combination according to the clinical characteristics of ESCC patients, and the optimal duration of postoperative immunoadjuvant therapy, etc., to achieve individualized and precise treatment for ESCC patients, and to achieve “better efficacy, fewer adverse reactions, and longer survival time” still remain the greatest challenges in the treatment of locally advanced esophageal squamous cell carcinoma at present.
